# Nutritional Impairment and Muscle Health in MASLD: The Potential Role of Eating Behaviors

**DOI:** 10.3390/jcm15218306

**Published:** 2026-10-25

**Authors:** Ioana Armașu, Maria-Christina Ungureanu, Liliana Gheorghe, Gina Eosefina Botnariu, Alexandru Florin Florescu, Anca Matei, Irina Jari, Roxana Elena Rusu, Elena Cojocaru, Ioana Vasiliu, Cristian Cojocaru, Letiția Leuștean

**Affiliations:** Grigore T. Popa University of Medicine and Pharmacy, 700115 Iași, Romania; ioana.armasu@umfiasi.ro (I.A.);

**Keywords:** eating behavior, MASLD, malnutrition, muscle health, sarcopenic obesity, GLP-1 receptor agonists

## Abstract

Obesity can conceal nutritional impairment and compromised muscle health in metabolic dysfunction-associated steatotic liver disease (MASLD). This critical narrative review examines the potential role of eating behaviors in nutritional impairment and muscle health in MASLD, focusing on adults with overweight or obesity while considering the limited findings in lean MASLD and, separately, pediatric evidence. Altered eating behaviors are associated with prevalent MASLD, but their contribution to malnutrition, myosteatosis, and sarcopenic obesity remains unproven. Nutritionally adverse dietary patterns and appetite loss may increase vulnerability, although evidence linking these factors to muscle outcomes derives predominantly from aging and general obesity populations. Total daily protein intake is better supported than specific meal-level distribution targets, while resistance exercise offers more consistent functional benefits than protein supplementation alone in the limited direct MASLD evidence. Restricting eating windows has not demonstrated additional hepatic benefit over usual care. Incretin-associated lean-mass loss does not by itself establish sarcopenia or disproportionate muscle harm and requires interpretation alongside muscle strength and function. Physical inactivity, socioeconomic circumstances, multimorbidity, and reverse causation complicate attribution to eating behavior. These findings support assessment beyond body mass index, integrating eating behaviors, nutritional risk, and muscle health. We propose an adult assessment framework; however, prospective studies are needed to validate it and clarify the independent contribution of eating behaviors to nutritional and muscle deterioration in MASLD.

## 1. Introduction

Obesity does not exclude malnutrition. In metabolic dysfunction-associated steatotic liver disease (MASLD), excess adiposity may coexist with inadequate nutrient intake, low muscle strength, reduced muscle quantity, and myosteatosis [1,2]. Guidelines emphasize lifestyle and nutritional care, while recognizing that body weight alone does not establish nutritional or muscle health [3,4,5,6,7]. Heterogeneous cardiovascular risk assessment further supports evaluation beyond body mass index (BMI) and liver-focused measures [8].

Malnutrition, sarcopenia, sarcopenic obesity, and myosteatosis overlap but are not interchangeable. The European Society for Clinical Nutrition and Metabolism/European Association for the Study of Obesity (ESPEN/EASO) consensus and the international clinical nutrition call to action clarify sarcopenic-obesity assessment [1,9]. The Global Leadership Initiative on Malnutrition (GLIM) criteria allow diagnosis at elevated BMI when a phenotypic and an etiologic criterion coexist [10,11]. These frameworks identify impairment without explaining the contribution of eating behavior.

MASLD studies report binge-eating symptoms, cognitive restraint, night eating, and broader eating-behavior scores [12,13,14,15]. Separate obesity and aging studies associate protein distribution, ultra-processed foods, and appetite loss with muscle outcomes [16,17,18]. Whether these observations form a causal pathway to malnutrition or sarcopenic obesity is unknown.

We distinguish direct clinical observations, extrapolation from related populations, and proposed mechanisms. This complements recent consensus work and reviews of incretin-associated nutritional risk [9,19,20]. The primary synthesis concerns adults with overweight or obesity. Pediatric evidence is discussed separately; lean MASLD and decompensated cirrhosis are not evaluated as target populations.

## 2. Methods

### 2.1. Search Strategy

The original literature cutoff was 31 July 2026. This narrative review draws on PubMed/MEDLINE-indexed reports and reference lists. Search concepts covered MASLD, metabolic dysfunction-associated steatohepatitis (MASH), non-alcoholic fatty liver disease (NAFLD), obesity, malnutrition, sarcopenia, muscle mass and quality, myosteatosis, eating behavior, binge eating, cognitive restraint, night eating, meal timing, protein intake, ultra-processed foods, and appetite loss. Glucagon-like peptide-1 (GLP-1) receptor agonists and glucose-dependent insulinotropic polypeptide (GIP)/GLP-1 agonists were considered for treatment-related outcomes. During revision, selected primary reports and reference lists were checked to refine the adult synthesis and add pediatric context. Physical activity, sedentary behavior, socioeconomic position, and multimorbidity were appraised as competing explanations. Exact search strings and screening counts are not reported, so this selection should not be interpreted as an exhaustive systematic search.

### 2.2. Eligibility Criteria

Clinical reports were selected for an eating-related exposure, a nutritional or muscle outcome, or a relevant MASLD/NAFLD population. Designs included randomized trials, observational studies, systematic reviews, and meta-analyses. Guidelines and consensus statements supplied diagnostic definitions. Named psychometric instruments were distinguished from self-reported dietary characteristics; non-standardized measures were retained with explicit limitations rather than treated as validated behavioral assessments.

The primary synthesis excludes exclusively pediatric populations, other primary causes of steatosis, and purely preclinical studies. Mechanistic sources are discussed separately in Section 6. Pediatric reports are presented in Section 4.4, outside the adult evidence matrix. Some source-defined adolescent cohorts include participants aged 18–19 years; their age ranges are retained rather than interpreted as an adult subgroup. Conference abstracts and preprints are not used as clinical evidentiary sources.

### 2.3. Study Selection and Evidence Grading

Evidence was appraised narratively by design, population, measurement, outcomes, and alternative explanations. Prespecification and independent duplicate selection are not documented. No registered protocol, formal risk-of-bias assessment, or quantitative synthesis is reported. SANRA (Scale for the Assessment of Narrative Review Articles) informs reporting [21]. Findings are labeled as direct clinical evidence, indirect evidence from related populations, or mechanistic/preclinical evidence.

### 2.4. Population and Scope

The primary population is adults with MASLD and overweight or obesity, using BMI thresholds of at least 25 kg/m^2^ in European populations or 23 kg/m^2^ in Asian populations. Mixed-adiposity cohorts are identified as general MASLD evidence, not obesity-specific results. Lean MASLD is not evaluated sufficiently for population-specific conclusions; a normal BMI establishes neither adequate nutrition nor preserved muscle. Decompensated cirrhosis is outside the target population because hepatic failure, portal hypertension, and altered metabolism introduce distinct nutritional risks. Transplant and geriatric evidence is retained only with source-population caveats. Pediatric disease differs in growth, puberty, histology, and family feeding context [22,23]. It is discussed separately in Section 4.4. Historical NAFLD populations are described using their original terminology where current MASLD eligibility was not individually established.

## 3. Conceptual Boundaries: What Is Being Measured?

Malnutrition is diagnosed after a positive validated nutrition-risk screen and requires at least one GLIM phenotypic criterion (non-volitional weight loss, low BMI, or reduced muscle mass) plus one etiologic criterion (reduced intake or assimilation, or disease burden and inflammation) [10,11]. Sarcopenia prioritizes low muscle strength, with low muscle quantity or quality confirming the diagnosis under the revised European Working Group on Sarcopenia in Older People criteria (EWGSOP2) [24]. Sarcopenic obesity requires both altered body composition, namely excess adiposity together with reduced skeletal muscle mass, and altered skeletal muscle function under the ESPEN/EASO framework; neither component is sufficient on its own [1]. Myosteatosis describes pathological fat infiltration and reduced muscle radiodensity; it can occur before low muscle quantity or overt functional decline [25]. Table 1 summarizes these boundaries.

Eating-related exposures comprise three classes. Eating behaviors, including binge eating, cognitive restraint, emotional eating, and night-eating syndrome, are assessed psychometrically or against diagnostic criteria [26,27,28]. Dietary-pattern characteristics, including protein distribution, processing level, and meal timing, are measured through dietary assessment. Appetite-related vulnerability concerns a reduced drive to eat; anorexia of aging is one example [29]. These classes have different reference standards and should not be combined into an unvalidated composite.

**Figure 1:**
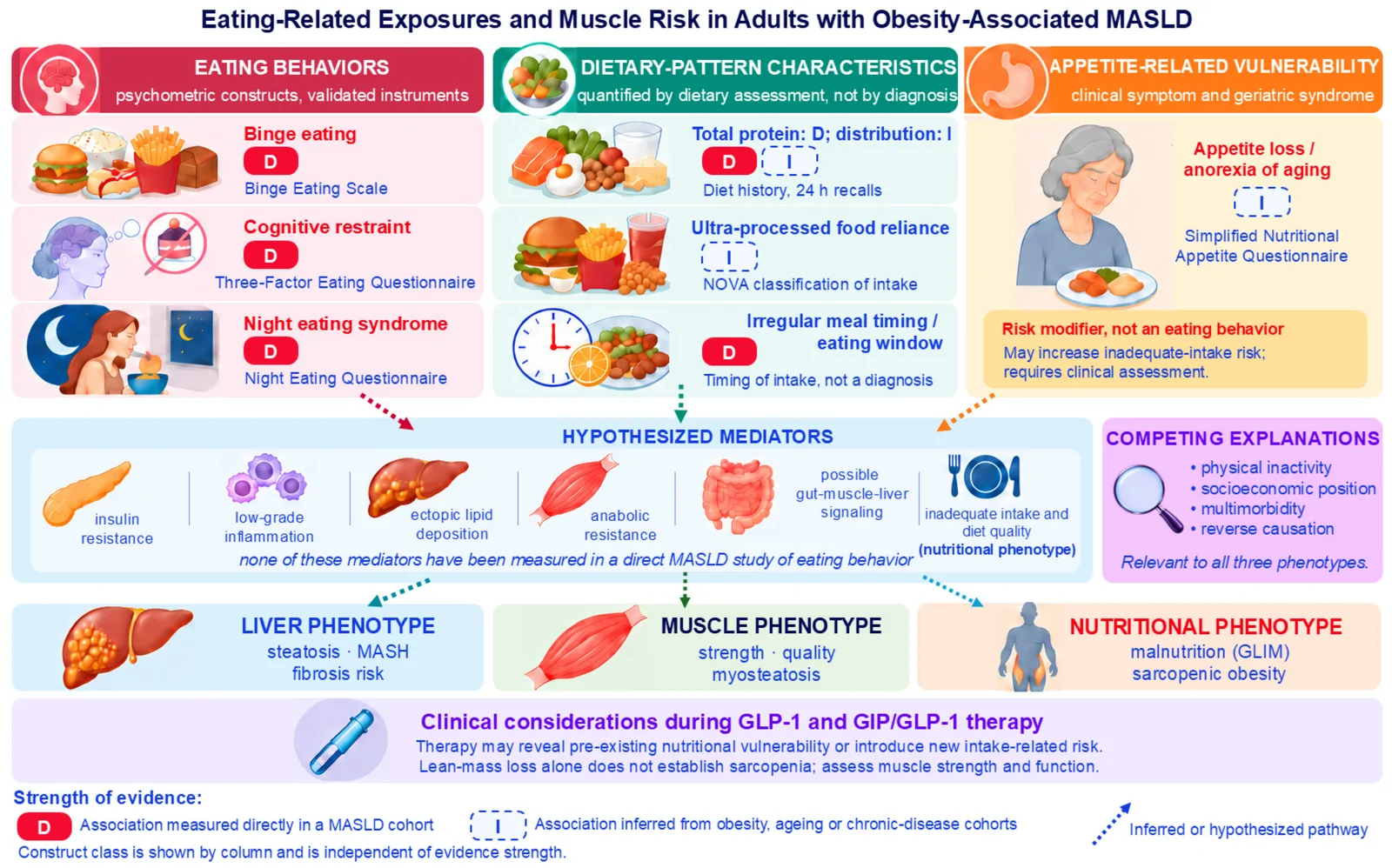
Eating-related exposures and nutritional or muscle risk in adults with obesity-associated MASLD. Columns identify construct class; D and I denote direct and indirect evidence. Direct evidence in a MASLD population does not necessarily establish muscle outcomes or causality. Total protein amount has limited direct trial evidence, whereas distribution has indirect evidence. Dashed connections indicate hypothesized pathways, not established causal relationships. Competing explanations are relevant to all three phenotypes. GLP-1 and GIP/GLP-1 therapy considerations are shown as a separate clinical note, not as a causal pathway. The figure does not establish causality or pediatric applicability.

We use the neutral term eating behaviors for the field and name each exposure separately. An umbrella label of disordered eating would pathologize cognitive restraint, which is not inherently disordered [26], and blur the distinction between night-eating syndrome and meal timing [27,28]. Table 1 and Figure 1 therefore organize exposures by construct class, independently of evidence strength.

## 4. Direct Evidence Linking Eating Behavior, MASLD, and Muscle Health

Table 2 presents the seven selected adult primary studies. The two randomized trials manipulate eating-window timing or protein intake with resistance training; they do not directly test the proposed behavior-to-malnutrition pathway.

### 4.1. Binge Eating and Broader Behavioral Profiles

In 100 Canadian MASLD outpatients, 20% had moderate-to-severe binge-eating symptoms, using a Binge Eating Scale threshold of 18; 31% had anxiety symptoms and 16% depressive symptoms. Poor Mediterranean-diet adherence affected 56%, and about one third reported low physical activity [13]. Nutritional status and muscle were not measured, and co-occurring behavioral, psychological, and activity factors were not disentangled.

Among 322 male Japanese graduate students, 11% had MASLD; total eating-behavior scores were higher in that group (median 102 versus 90), particularly perceptions of constitution and weight [14]. The median age of 22 years, single-center setting, ultrasonographic diagnosis, and locally used instrument limit generalizability. Neither study demonstrates a nutritional or muscle consequence.

### 4.2. Cognitive Restraint and Lean Mass

Among 138 liver-transplant recipients assessed at least 12 months after transplantation, 26.1% had MASLD. Lower percentage lean mass (OR 0.926, 95% CI 0.881–0.972) and higher cognitive restraint (OR 1.021, 95% CI 1.005–1.036) were independently associated with MASLD after adjustment for age, sex, and time since transplantation [12]. The study did not demonstrate an association between restraint and lean mass.

This observation is hypothesis-generating. Temporality and mediation were not tested; restraint may follow weight gain rather than cause it. Its per-point association is small, with uncertain clinical meaning. BIA is hydration-dependent, while immunosuppression, corticosteroids, pre-transplant impairment, and weight regain introduce confounding specific to transplantation. The findings cannot substitute for studies in non-transplant MASLD.

### 4.3. Night Eating, Irregular Timing, and Circadian Behavior

In 93 adults with biopsy-proven MASLD, night-eating syndrome was recorded in 22 patients with a non-morning chronotype and nine with a morning chronotype: 23.7% and 9.7% of the whole cohort, not within-group prevalences. Neither night eating nor chronotype was associated with histologic severity; age and poor sleep quality predicted advanced fibrosis [15]. In Guatemala, 33 ultrasound-confirmed MASLD cases were compared with 43 controls. In the behavioral sub-sample of 60, regular meal timing was associated with lower MASLD odds (OR 0.12, 95% CI 0.02–0.64) [30]. Only one control underwent ultrasound, and the letter does not describe a validated meal-timing instrument, limiting interpretation. In a 12-week trial of 197 adults with overweight or obesity, early, late, and self-selected eight-hour eating windows did not improve hepatic fat, liver markers, or microbiota beyond usual Mediterranean-diet care [31]. Within-group improvements accompanied weight loss. These heterogeneous exposures support further study, not a common independent mechanism.

Direct adult evidence supports behavioral associations with prevalent MASLD. It does not establish progression from a behavioral phenotype to malnutrition, myosteatosis, or sarcopenic obesity.

### 4.4. Pediatric and Adolescent MASLD, Reported Separately

Table 3 provides pediatric context outside the adult synthesis and evidence matrix. Historical NAFLD and non-alcoholic steatohepatitis (NASH) studies are discussed for their relevance to MASLD; participants were not individually reclassified using current criteria. No pediatric estimate is pooled with an adult result.

In 159 adolescents assessed before bariatric surgery (mean age 16.4 years; BMI 53.7 kg/m^2^), binge-eating disorder (BED) symptomatology and internalizing psychopathology were associated with biopsy-defined NAFLD [33]. Generic binge eating alone was not significantly associated. In a UK case–control study, 24 children with NAFLD had higher restrained-eating scores than eight controls with obesity after BMI z-score adjustment; dietary differences did not survive adjustment and under-reporting was common [34]. A study of 38 patients aged 5–19 years linked fructose intake to insulin resistance and lower adiponectin [35]. These studies measured different behavioral or dietary constructs, not a shared syndrome.

Nocturnal eating was inversely associated with borderline/definite NASH within the selected surgical cohort [33]. This is not evidence of protection. Together with the null adult biopsy result [15], it does not establish a causal role in liver injury; nocturnal eating and eating-window interventions [31] should not be equated. Anorexia of aging is inapplicable to children, but other forms of appetite-related vulnerability are not thereby excluded.

MRI (magnetic resonance imaging) height-indexed psoas area was associated with steatosis severity in 100 biopsy-assessed patients and with hepatic proton density fat fraction (PDFF) in a subgroup of 95; it was not associated with stiffness in the imaging cohort of 263 [36]. These groups overlap. A systematic review of sarcopenic obesity reported prevalence of 5.66–69.7% in girls and 7.2–81.3% in boys, with non-standardized definitions [37]. Growth, puberty, and measurement differences preclude automatic extrapolation between pediatric and adult populations. None of these studies establish an eating-behavior-to-malnutrition-to-muscle pathway or validate adult diagnostic criteria for children.

## 5. Nutritionally Adverse Patterns: Evidence and Limits

### 5.1. Protein Amount, Quality, and Distribution

Protein dose and leucine availability influence muscle protein synthesis in older adults [16,38], but total daily intake is better supported than a universal per-meal target. ESPEN recommends at least 1.0–1.2 g/kg/day for healthy older adults and 1.2–1.5 g/kg/day during illness [39]. Higher intakes have been proposed during weight loss in older adults with obesity [40]. These are not MASLD-specific targets; kidney function, age, activity, energy restriction, and the choice of reference weight require individual consideration.

In a four-week trial, 45 adults with MASLD under approximately 30% energy restriction received higher protein, resistance training, both, or neither [32]. Higher-protein groups received 1.5 g/kg/day total protein, approximately 0.8 from food and 0.7 from whey; comparison groups received approximately 0.8 g/kg/day. No significant BIA-estimated muscle-mass change was detected, including without training; this does not establish long-term preservation. Peak torque and total work improved across most regions with training and fewer with protein alone. The trial supports functional assessment and resistance exercise, but does not isolate the mechanism behind the modest additional protein effect.

The often-quoted 25–30 g per meal derives from acute anabolic studies in aging populations, not MASLD outcome trials. Protein plans should therefore specify individualized daily intake rather than prescribe an unvalidated meal-level threshold.

### 5.2. Ultra-Processed Foods and Dietary Quality

Greater ultra-processed food intake is associated with frailty, sarcopenia, and low strength in older adults [18,41]. A prospective cohort linked healthier dietary patterns with less progression to sarcopenic obesity [42]. Protein and micronutrient displacement and inflammatory effects are plausible explanations, but no cited MASLD study isolates this exposure from activity, socioeconomic position, energy intake, or multimorbidity.

### 5.3. Appetite Loss and Age-Related Vulnerability

Anorexia of aging reflects sensory changes, altered gut signaling, oral disease, medication effects, inflammation, and psychosocial factors. It is associated with malnutrition and mortality [17,43]. In MASLD it is most relevant to older or multimorbid patients, not a general eating-behavior mechanism.

### 5.4. Competing Explanations: Physical Activity, Socioeconomic Position, Multimorbidity, and Reverse Causation

Four competing explanations must be considered before attributing muscle impairment to eating behavior.

Physical inactivity can affect both intake patterns and muscle health. A fatty-liver cohort reported sarcopenia in 40.7% versus 18.5% of controls, with activity among the associated health metrics [44]. A Canadian MASLD cohort also illustrated co-occurring binge-eating symptoms and low activity without analytical separation [13]. Activity and sedentary time should be measured alongside diet, preferably objectively.

Socioeconomic position influences food access, preparation, work schedules, and health literacy; it is associated with diet quality and malnutrition in older adults [45]. Income or education alone may incompletely represent this confounding.

Multimorbidity and medication burden can alter appetite, gastrointestinal tolerance, activity, and muscle. In 499,046 UK Biobank participants, multimorbidity was associated with probable sarcopenia (OR 1.96, 95% CI 1.91–2.02) [46]. This adjacent-population association should not be presented as a MASLD-specific effect.

Reverse causation is plausible: reduced function may change appetite, energy needs, shopping, and meal preparation. The selected studies do not establish that behavioral exposure precedes nutritional or muscle impairment.

Intervention effects also require separation. A meta-analysis of 37 MASLD trials reported weight reduction with a Mediterranean diet (−2.38 kg), aerobic exercise (−1.56 kg), and combined training (−1.90 kg), with the largest alanine aminotransferase reduction after resistance training [47]. These comparisons do not identify baseline eating behavior as a response modifier.

Table 4 summarizes the adult evidence across the three exposure classes, highlighting source populations and the limits of inference for nutritional and muscle outcomes in MASLD.

## 6. Plausible Biological Links: From Eating Behavior to Liver and Muscle

Insulin resistance, ectopic lipid deposition, inflammation, and mitochondrial dysfunction connect hepatic and muscle biology [2,48]. Irisin is a candidate signal, not an established MASLD treatment [49]. Myosteatosis may precede reduced muscle quantity [25], and MASLD cohorts associate adverse muscle phenotypes with fibrosis or metabolic risk [50,51,52,53]. Reviews emphasize heterogeneous definitions and predominantly cross-sectional evidence [54,55,56]. These links do not establish an eating-behavior mediator.

Microbial composition, short-chain fatty acids, permeability, bile acids, and inflammation offer a possible gut–muscle–liver pathway [57,58]. Much human evidence concerns cirrhosis or general sarcopenia. No validated microbial signature identifies the proposed phenotype, and eating-window restriction did not alter microbiota beyond usual care in the cited trial [31].

Figure 1 separates measured associations from inferred mechanisms. Every mediator connection is dashed because no selected direct eating-behavior study tested mediation.

## 7. Clinical Assessment in Adults: Screening and Diagnosis

Separate nutritional risk, eating behavior, muscle function, composition, and diagnosis. GLIM follows a positive setting-appropriate screen, such as the Malnutrition Screening Tool (MST), Nutritional Risk Screening 2002 (NRS-2002), or Mini Nutritional Assessment Short Form (MNA-SF) [10,11]. Diagnosis requires a phenotypic and an etiologic criterion. Obesity neither excludes malnutrition nor establishes it.

SARC-F covers strength, walking assistance, chair rise, stairs, and falls. Its low sensitivity means a negative result should not prevent objective testing in high-risk patients [59,60]. Older age, advanced fibrosis, rapid weight loss, low appetite, impaired mobility, hospitalization, multimorbidity, and potent weight-loss therapy support assessment of grip strength or chair rise and, where indicated, body composition.

Dual-energy X-ray absorptiometry (DXA) quantifies lean tissue, not myosteatosis. Bioelectrical impedance analysis (BIA) and phase angle depend on hydration and equations [61]. Computed tomography (CT) or magnetic resonance imaging (MRI) can assess muscle area, fat infiltration, and adiposity [62,63]. Opportunistic CT avoids additional radiation only when an existing scan is reused. Thresholds require population- and modality-specific validation [64]. Table 5 summarizes these adult assessments.

Name the behavioral instrument and interpretation. Options include the Binge Eating Scale, the Three-Factor Eating Questionnaire for restraint, uncontrolled eating, and emotional eating [26], and the Night Eating Questionnaire interpreted against published criteria [27,28,65]. A symptom score does not replace diagnostic assessment. Diet history or repeated recalls assess intake quality, protein distribution, and timing. Measure activity alongside these domains; no composite MASLD eating-behavior score is validated.

## 8. Incretin-Based Therapy: Revealing and Generating Nutritional Vulnerability

### 8.1. Hepatic Benefit and What Lean Mass Means

Incretin therapies improve weight, glycemia, and hepatic outcomes. Semaglutide improved MASH resolution and fibrosis endpoints in ESSENCE [66]. These benefits remain central. Lean mass includes water and non-muscle tissues; its reduction is not synonymous with sarcopenia, which requires functional assessment.

### 8.2. How Much Lean Mass Is Lost, and Compared with What

Meta-analysis reports lower absolute lean mass but a higher lean proportion of body weight during GLP-1 therapy, with heterogeneity and limited functional data [67]. Clinical interpretation depends on baseline reserve, loss rate, diet, and exercise [19,68,69]. Function may remain stable or improve despite lower lean mass [70,71].

Distinguish absolute lean-mass change in kilograms, lean tissue as a proportion of weight lost, and lean mass relative to body weight. These quantities can move in different directions.

One meta-analysis restricted to at least 10% weight loss (21 studies; 1334 participants) reported fat-free-mass changes of −1.8 kg (95% CI −2.6 to −1.0) with diet/exercise, −4.8 kg (−5.6 to −3.9) with incretin therapy versus placebo, and −9.1 kg (−12.3 to −6.0) after surgery. Fat-free tissue represented 14.9%, 33.3%, and 34.2% of weight lost; within lifestyle interventions, 22.3% without exercise versus 7.7% with exercise [72]. Another analysis (20 trials; 15,782 participants) reported lean proportions of 35.2% with semaglutide, 25.4% with tirzepatide, 26.8% with liraglutide, and 26.2% with lifestyle intervention, without a significant pharmacotherapy-lifestyle difference; lifestyle plus resistance training yielded 17.5% [73].

These analyses differ in inclusion thresholds, comparators, exercise co-interventions, and measurement methods, limiting direct comparison. A network meta-analysis of 22 trials (2258 participants) found a pooled −0.86 kg lean-mass change, approximately 25% of weight lost, without change in relative lean mass [74]. Estimates are conditional on the quantity and methods assessed, not interchangeable drug-specific benchmarks.

The historical quarter-of-weight-loss rule varies with baseline adiposity, deficit, and loss rate [75]. In the SURMOUNT-1 DXA substudy (*n* = 160; 72 weeks), tirzepatide reduced weight, fat mass, and lean mass by 21.3%, 33.9%, and 10.9%, versus 5.3%, 8.2%, and 2.6% with placebo. Both groups lost approximately 75% fat and 25% lean tissue [76]. Similar proportions do not imply similar absolute losses or functional consequences.

### 8.3. Is the Effect Dose-Dependent?

The identified literature does not establish dose-dependent muscle harm in MASLD. SURMOUNT-1 pooled tirzepatide at doses of 5, 10, and 15 mg, precluding dose-specific body-composition inference [76]. Cross-agent network comparisons confound dose with population and duration [74].

The network analysis placed 15 mg weekly of high-potency tirzepatide and 2.4 mg weekly of semaglutide among agents with greater lean-mass loss. Liraglutide at a dose of 3.0 or 1.8 mg daily reduced weight without a significant lean-mass reduction in that analysis [74]. Direct dose-ranging composition data come from investigational retatrutide in type 2 diabetes, not MASLD. Its substudy enrolled 189 participants across retatrutide, placebo, and dulaglutide arms; 103 completed both DXA scans. At 36 weeks, fat mass fell by 4.9%, 15.2%, 26.1%, and 23.2% with retatrutide doses of 0.5, 4, 8, and 12 mg. The lean proportion of weight lost did not increase with dose [77]. Regression-derived estimates, incomplete scans, the non-monotonic fat response, and absent functional testing limit inference. This is absence of a detected disproportionate lean-tissue signal, not proof that one cannot occur.

Higher potency may produce greater absolute lean loss through greater overall weight loss. The available evidence does not justify reducing dose solely to protect muscle. MASLD dose-stratified studies should assess composition and function, particularly in patients with low baseline reserve.

### 8.4. Does Muscle Function Change?

Strength and performance are measured less often than composition.

In a one-year maintenance trial, exercise plus liraglutide best maintained weight loss [78]; relative strength rose 3.3% versus a 7.8% fall with placebo, with the benefit attributed to exercise [79]. In the single-arm SLIM LIVER trial (51 adults with human immunodeficiency virus (HIV) and MASLD; 24 weeks), psoas volume fell 9.3%, fat fraction was unchanged, and gait/chair-rise changes were non-significant; slow gait prevalence fell from 63% to 46% [80]. SURPASS-3 MRI showed lower muscle volume with reduced fat infiltration, illustrating different compositional responses [81].

Countervailing observational evidence in older adults with diabetes reports greater muscle and strength loss over 24 months [82] and markers of neuromuscular-junction degradation [83]. These non-MASLD findings do not establish causality but support monitoring in patients with low reserve.

A MASLD systematic review included 12 studies and 810 participants; three studies, comprising 477 participants, reported strength without detected deterioration despite weight loss. Four assessed muscle quality, generally preserved or improved [84]. Thus, direct functional evidence does exist, but heterogeneous methods and limited follow-up do not exclude longer-term harm.

### 8.5. Revealing Versus Generating Vulnerability

Therapy may reveal low pre-existing reserve or generate intake-related risk through appetite suppression, nausea, vomiting, food aversion, and rapid energy restriction [20,85,86]. No cited prospective study establishes a pre-treatment eating-behavior phenotype as a predictor of clinically important muscle loss. Baseline reserve and functional change deserve attention alongside hepatic and weight benefits.

### 8.6. Clinical Implication

Before and during treatment, assess weight trajectory, appetite, gastrointestinal symptoms, dietary adequacy, strength, and relevant eating-behavior symptoms. Provide dietetic and resistance-exercise support according to risk. Expected DXA lean loss alone should not discourage indicated treatment or trigger dose reduction.

## 9. Proposed Non-Validated, Risk-Stratified Assessment in Adults

**Figure 2:**
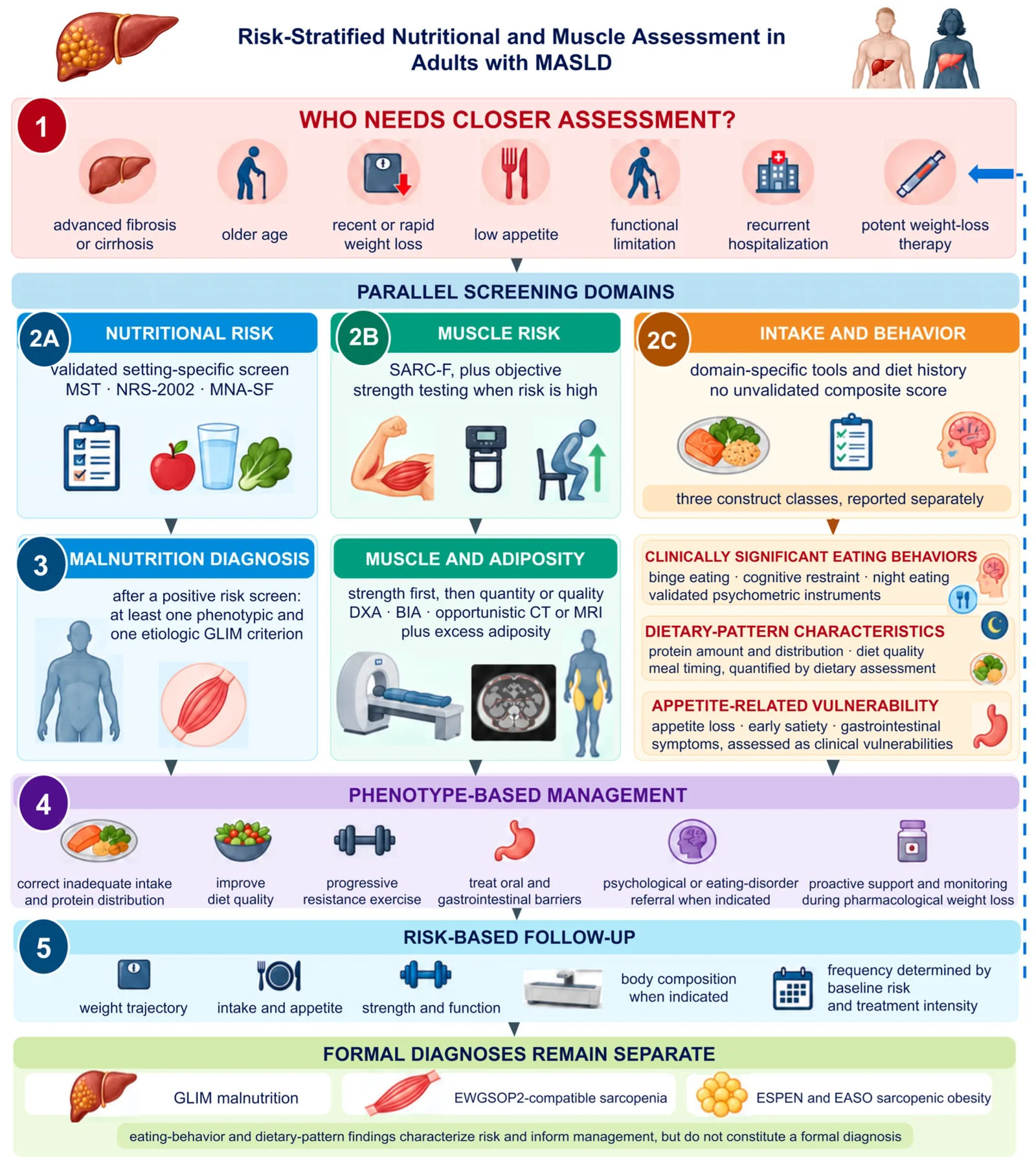
Proposed non-validated adult assessment framework. Nutrition-risk screening precedes GLIM; SARC-F is not the sole gateway in high-risk patients. Eating behaviors, dietary characteristics, and appetite symptoms characterize risk rather than constitute diagnoses. Numbered labels indicate assessment steps, with domains 2A–2C assessed in parallel.Solid connections show the proposed assessment sequence; dashed connections indicate re-screening. Colors distinguish domains and stages of assessment and management; shapes and font colors do not indicate risk severity or evidence strength. Illustrations are schematic. Pediatric applicability is not established.

Figure 2 is an adult conceptual framework, not a validated algorithm. Advanced fibrosis, older age, unintentional or rapid loss, low appetite, impaired function, hospitalization, or potent weight-loss therapy prompt parallel nutritional and muscle assessment. GLIM follows validated risk screening; high-risk patients receive objective strength assessment despite negative SARC-F. Behavioral, dietary, and appetite domains remain separate.

Formal diagnoses remain distinct: GLIM malnutrition, EWGSOP2-compatible sarcopenia, and ESPEN/EASO sarcopenic obesity. Management may address inadequate intake, diet quality, exercise, oral/gastrointestinal barriers, and psychological needs. Follow-up reflects baseline risk and treatment intensity. Adult criteria, screening tools, and protein recommendations are not proposed for pediatric use.

The proposed risk-stratified assessment framework for adults with MASLD is summarized in Figure 2. This framework is conceptual and has not been validated.

## 10. Research Priorities

Prospective cohorts should measure validated eating-behavior constructs, diet, activity, muscle strength and composition, fibrosis, and medication exposure together. This permits temporal and mediation analyses and testing of distinct exposure phenotypes. Incretin trials should assess strength and performance alongside imaging and investigate baseline behavioral modifiers.

Muscle-area, radiodensity, BIA, and phase-angle thresholds require validation across sex, age, ethnicity, and fibrosis. Microbiome studies should test mediation rather than parallel associations. Trials should separate effects of weight loss, diet quality, meal timing, and exercise.

One feasible approach is repeated adult assessment at baseline, 12, and 24 months using domain-specific questionnaires [65], dietary records, objective measures of physical activity and sedentary time, grip/chair-rise tests, and consistent muscle imaging. Socioeconomic position, comorbidity, and medication burden should be prespecified covariates [45,46]; sample size requires justification for the intended outcomes. Nested dose-stratified trial substudies could pair composition with function [76]. Parallel lean and pediatric strata need their own definitions rather than automatic transfer of the adult framework. In historical non-obese NAFLD, imaging-defined low muscle mass and myosteatosis were associated with disease independently of visceral adiposity [87]; this is not equivalent to clinically diagnosed sarcopenia in lean MASLD. Diet quality is inversely associated with lean MASLD in population data [88], but the present review does not establish corresponding behavioral or muscle pathways.

## 11. Limitations

This review was conceived as a critical narrative synthesis rather than a systematic review. A registered protocol, documented duplicate screening, formal risk-of-bias assessment, and quantitative synthesis were not included in the reported methodology, which may affect reproducibility. The seven adult studies discussed provide a selected perspective on the evidence rather than an exhaustive inventory.

The search focused on PubMed/MEDLINE, and the appraised reports were in English. Other bibliographic databases, including PsycINFO, as well as gray literature and study registries, were not systematically searched. Relevant evidence in other languages or unpublished sources may therefore not be fully represented. Revision-stage source checks were intended to verify the cited evidence rather than to provide a systematic literature update. The meal-timing study was reported as a research letter, with limited detail on ascertainment methods [30].

The adult evidence comprises four cross-sectional studies, one case–control study, and two short intervention trials. These studies inform associations and short-term outcomes but do not establish whether eating-behavior phenotypes precede incident malnutrition, sarcopenic obesity, or myosteatosis. Reverse causation and the competing explanations discussed in Section 5.4 remain possible interpretations of the observed relationships.

Variation in exposure instruments, cut-points, and reference periods should be considered when comparing findings. DXA-derived lean tissue, CT-based muscle area and radiodensity, and BIA estimates capture related but distinct aspects of body composition and are not interchangeable. Findings from specialized transplant cohorts and from aging or general obesity populations also require care when extrapolated to broader MASLD populations.

Pediatric observations were considered separately to provide context rather than a comprehensive pediatric synthesis. Selected surgical populations, small samples, growth-related measurement differences, and non-standardized muscle definitions warrant cautious interpretation. These observations should not be taken as validation of the proposed adult assessment framework for use in children.

## 12. Conclusions

Eating behavior is a plausible and clinically relevant, but as yet unproven, link between adult obesity-associated MASLD and nutritional or muscle impairment. Direct evidence is limited and heterogeneous, and the reviewed studies do not establish progression from eating-behavior symptoms to malnutrition, myosteatosis, or sarcopenic obesity. Findings from transplant, aging, and general obesity populations require caution when applied to broader MASLD populations.

Total daily protein intake is better supported than fixed meal-level targets, although short-term MASLD evidence does not establish long-term muscle preservation. Incretin-associated lean-mass loss should be interpreted alongside muscle strength and function, rather than equated with sarcopenia or disproportionate muscle harm. Longer-term studies with functional endpoints are needed to clarify these outcomes.

These findings support assessment beyond BMI, integrating eating-behavior symptoms, nutritional risk, and risk-based muscle testing. The proposed adult assessment framework requires prospective validation, while pediatric observations provide context without validating adult criteria or a life-course causal pathway.

Overall, nutritional and muscle health in MASLD should be considered beyond body weight, while the independent contribution of eating behaviors warrants further investigation.

## Figures and Tables

**Table 1:** Distinct constructs relevant to adult obesity-associated MASLD.

Construct	Core Definition/Framework	Principal Clinical Implication
Malnutrition	Positive validated risk screen followed by ≥1 GLIM phenotypic and ≥1 etiologic criterion [10,11]	Can occur at elevated BMI; not synonymous with sarcopenia
Sarcopenia	Low strength indicates probable disease; low muscle quantity/quality confirms EWGSOP2 [24]	Function is central; lean-mass loss alone is insufficient
Sarcopenic obesity	Excess adiposity plus impaired muscle mass/function under ESPEN/EASO [1]	Requires assessment of both adiposity and muscle
Myosteatosis	Pathological intramuscular/intermuscular fat or reduced muscle radiodensity on cross-sectional imaging [25]	May indicate poor muscle quality despite preserved quantity
Eating behavior (psychometric construct)	Binge eating, cognitive restraint, emotional eating, night-eating syndrome; assessed with a validated psychometric instrument or against published diagnostic criteria [26,27,28]	A clinical finding in its own right; the instrument and cut-point must be named
Dietary-pattern characteristic	Protein amount and distribution across meals, degree of food processing, timing of meals; measured by dietary assessment, not by psychometrics	A property of intake rather than a behavior; should not be labeled as disordered eating
Appetite-related vulnerability	Anorexia of aging: multifactorial reduction in the drive to eat, related to sensory and gut-hormone signaling, oral disease, medication burden, and inflammation [29]	A risk modifier that amplifies the other classes; most consequential during appetite-suppressing therapy

**Table 2:** Selected adult primary studies of eating-related exposures in MASLD.

Study, Year, Design	Population (*n*)	Exposure and Instrument	Principal Finding and Main Limitations
Cardoso et al., 2025; cross-sectional [12]	Liver-transplant recipients at least 12 months post-transplant (*n* = 138); 26.1% with post-transplant MASLD	Three-Factor Eating Questionnaire-R21; bioelectrical impedance; 24 h dietary recall	MASLD associated with greater adiposity, lower percentage lean mass (OR 0.926, 95% CI 0.881–0.972), and higher cognitive restraint (OR 1.021, 95% CI 1.005–1.036). Limitations: Transplant-specific population and immunosuppression; hydration-dependent body-composition method; no mediation or temporality; small per-point effect size
Robertson et al., 2026; cross-sectional [13]	Adults with MASLD attending a single Canadian hepatology center (*n* = 100); 27 with advanced fibrosis	Binge Eating Scale (cut-point 18); energy-restricted Mediterranean Diet Adherence Screener; International Physical Activity Questionnaire Short Form; Hospital Anxiety and Depression Scale	A total of 20% with moderate-to-severe binge eating; 31% anxiety and 16% depressive symptoms; 56% poor Mediterranean adherence; approximately one third with low physical activity. Limitations: No nutritional or muscle outcome measured; behavior and physical inactivity not separated analytically
Miwa et al., 2024; cross-sectional [14]	Male graduate students at a single Japanese university (*n* = 322); 11% with MASLD, median age 22 years	Eating Behavior Questionnaire of the Japan Society for the Study of Obesity; hepatic steatosis on ultrasonography	Higher total eating-behavior score in MASLD (median 102 versus 90 points, *p* = 0.006); perception of constitution and weight the strongest contributing category. Limitations: Narrow demographic, single center, ultrasonographic outcome, instrument not in routine international use; no muscle or nutritional outcome
Kani et al., 2023; cross-sectional [15]	Patients with biopsy-proven MASLD (= 93)	Morningness-Eveningness Questionnaire; Pittsburgh Sleep Quality Index; Night Eating Questionnaire	Night-eating syndrome recorded in 22 of 93 patients with a non-morning chronotype and 9 of 93 with a morning chronotype (23.7% and 9.7% of the whole cohort, *p* = 0.001); neither night eating nor chronotype associated with histologic severity or fibrosis; advanced fibrosis predicted by age and poor sleep quality. Limitations: Single center, cross-sectional, no muscle or nutritional outcome; percentages reported against the whole cohort rather than within chronotype groups; negative for the hepatic endpoint
Sguazzini et al., 2026; case–control research letter [30]	Adults attending three primary-care clinics in Guatemala (*n* = 76): 33 ultrasound-confirmed MASLD cases and 43 controls; behavioral sub-sample *n* = 60 (32 cases, 28 controls)	Self-reported regularity of meal timing; clustering of cardiometabolic risk factors	Regular meal timing associated with lower odds of MASLD (OR 0.12, 95% CI 0.02–0.64); knowledge OR 0.07 (0.01–0.51). Limitations: Research-letter format, ascertainment instrument not described, only one control imaged, wide intervals, no temporality or muscle outcome
Dote-Montero et al., 2026; randomized controlled trial [31]	Adults with overweight or obesity (*n* = 197), including a MASLD subgroup; 12 weeks	Early, late, or self-selected eight-hour time-restricted eating versus Mediterranean-diet usual care; hepatic fat fraction by magnetic resonance imaging; 16S rRNA fecal microbiota	No between-group difference in hepatic fat fraction, liver markers, or microbiota; within-group reductions tracked achievement of at least 5% weight loss. Limitations: Not restricted to MASLD, not powered for secondary outcomes, no muscle outcome; informative as a negative result for eating-window timing
Kim et al., 2026; randomized, double-blind controlled trial [32]	Adults with MASLD under approximately 30% caloric restriction (*n* = 45); four weeks	Total protein 1.5 g/kg/day (approximately 0.8 from food plus 0.7 from whey) versus approximately 0.8 g/kg/day; with or without supervised resistance training; bioelectrical impedance analysis (BIA) and isokinetic function	No significant skeletal-muscle-mass change detected over four weeks. Functional gains occurred across most regions with training and fewer with protein alone. Limitations: *n* = 45, short follow-up, BIA, substantial energy restriction, no hepatic endpoint; not evidence of long-term muscle preservation

Table abbreviations: OR, odds ratio; CI, confidence interval; BIA, bioelectrical impedance analysis; rRNA, ribosomal ribonucleic acid. Diagnostic and measurement labels reflect each original study.

**Table 3:** Pediatric evidence, separate from the adult synthesis.

Study, Year, Design	Population (n)	Exposure and Instrument	Principal Finding and Main Limitations
Ley et al., 2021; cross-sectional [33]	Pre-bariatric adolescents, ages 13–19 (*n* = 159); mean BMI 53.7 kg/m^2^; NAFLD/NASH assessed by liver biopsy.	Adapted items from Questionnaire on Eating and Weight Patterns-Revised, Night Eating Questionnaire, and Look AHEAD (Action for Health in Diabetes); youth self-report subgroup *n* = 98.	BED symptomatology and internalizing symptoms associated with NAFLD; nocturnal eating inversely associated with borderline/definite NASH. Selected severe-obesity cohort; cross-sectional; no muscle outcome.
Gibson et al., 2015; case–control [34]	Biopsy-defined NAFLD (*n* = 24) and obesity controls (*n* = 8); ages 8–18.	Dutch Eating Behavior Questionnaire; recalls, seven-day diaries, pedometry.	Higher restraint after BMI z-score adjustment; more steps; no adjusted dietary difference. Small control group; under-reporting 75% versus 87%; no muscle outcome.
Mager et al., 2010; cross-sectional [35]	Sonographic fatty liver, ages 5–19 (*n* = 38).	Dietary intake, activity, energy expenditure, and fat-free mass.	High fructose and low polyunsaturated-fat intake; fructose associated with insulin resistance and lower adiponectin. No control group; dietary, not psychometric, exposure.
Yodoshi et al., 2020; retrospective cohort [36]	Patients aged <20; histology *n* = 100, stiffness *n* = 263, PDFF subgroup *n* = 95; overlapping groups.	MRI psoas area/height squared; community deprivation as a covariate.	Lower psoas index associated with steatosis and PDFF, not stiffness. Retrospective, surrogate muscle measure; no function or eating exposure. Abstract and full-text sample descriptions differ.
Zembura and Matusik, 2022; systematic review [37]	Children and adolescents; 18 studies, not restricted to MASLD.	Sarcopenic obesity, variously defined	Prevalence: girls 5.66–69.7%, boys 7.2–81.3%. Heterogeneous thresholds prevent comparable or MASLD-specific prevalence estimates.

**Table 4:** Adult evidence matrix: construct, source population, and inference.

Exposure	Construct Class	Direct MASLD Evidence (Source Population)	Indirect Muscle or Nutrition Evidence	Bottom Line
Binge eating/broader behavior scores	Clinically significant eating behavior	Frequent symptoms and association with prevalent MASLD [13,14] (Canadian hepatology outpatients and Japanese male students; both cross-sectional [13,14])	No direct prospective MASLD muscle outcome	Worth identifying because it is treatable and affects adherence; no evidence that it causes malnutrition or muscle loss
Cognitive restraint	Clinically significant eating behavior	Associated with post-transplant MASLD; lower relative lean mass in same cohort [12] (Brazilian liver-transplant recipients; cross-sectional [12])	Cross-sectional, specialized population	Hypothesis-generating only: one specialized cohort, small per-point effect, no temporality or mediation
Night eating/irregular timing	Night eating: clinically significant behavior; irregular timing: dietary-pattern characteristic	Mixed observational findings; no histologic-severity association in biopsy cohort [15,30] (Turkish biopsy-proven MASLD cohort and a case–control study; randomized trial in adults with overweight or obesity [15,30,31])	No direct muscle endpoint; randomized eating-window trial negative beyond weight loss [31]	Not an established independent driver; the randomized evidence is negative beyond weight loss, and timing is better treated as an adherence variable
Protein amount; distribution	Dietary-pattern characteristic	Amount: four-week MASLD trial [32]; distribution: no direct MASLD outcome study	Distribution: acute anabolic and aging evidence [16,38]	Individualize daily protein; distribution remains clinically unvalidated in MASLD
Ultra-processed foods/low diet quality	Dietary-pattern characteristic	MASLD relevance supported indirectly by guideline and metabolic evidence [3,4] (guideline and metabolic evidence only; no MASLD-specific exposure study [3,4])	Associated with sarcopenia/frailty in older adults [18,41]	Consistent association in aging cohorts, never isolated from socioeconomic position or physical activity within MASLD
Appetite loss/anorexia of aging	Appetite-related vulnerability	No direct general-MASLD prospective evidence (no general-MASLD population)	Associated with malnutrition and mortality in older adults [17,43]	A risk modifier rather than an eating behavior; most consequential in older patients and during appetite-suppressing therapy

**Table 5:** Muscle and body-composition assessment in adults.

Modality	Principal Measurements	Main Limitation
Handgrip/chair-stand	Muscle strength and functional impairment	Influenced by pain, cognition, and technique; does not quantify mass
DXA	Appendicular lean mass, fat mass, bone mineral density	Lean tissue is not synonymous with skeletal muscle; no myosteatosis
BIA/phase angle	Estimated lean/fat mass; phase angle as cellular-integrity marker [61]	Hydration- and equation-dependent
CT (opportunistic)	Muscle area, radiodensity/myosteatosis, visceral and subcutaneous fat	Threshold heterogeneity; avoids additional radiation only when a scan performed for another indication is reused
MRI/MRI-PDFF	Muscle volume/fat infiltration, adipose distribution, hepatic fat	Cost, acquisition time, and limited availability

## Data Availability

No new data were created or analyzed in this narrative review. Data sharing is therefore not applicable.

## Abbreviations

AI, artificial intelligence; BED, binge-eating disorder; BIA, bioelectrical impedance analysis; BMI, body mass index; CI, confidence interval; CT, computed tomography; D, direct evidence; DXA, dual-energy X-ray absorptiometry; EASO, European Association for the Study of Obesity; ESPEN, European Society for Clinical Nutrition and Metabolism; EWGSOP2, revised European Working Group on Sarcopenia in Older People consensus; GIP, glucose-dependent insulinotropic polypeptide; GLIM, Global Leadership Initiative on Malnutrition; GLP-1, glucagon-like peptide-1; HIV, human immunodeficiency virus; I, indirect evidence; MASLD, metabolic dysfunction-associated steatotic liver disease; MASH, metabolic dysfunction-associated steatohepatitis; MEDLINE, Medical Literature Analysis and Retrieval System Online; MNA-SF, Mini Nutritional Assessment Short Form; MRI, magnetic resonance imaging; MRI-PDFF, magnetic resonance imaging-proton density fat fraction; MST, Malnutrition Screening Tool; NAFLD, non-alcoholic fatty liver disease; NASH, non-alcoholic steatohepatitis; NRS-2002, Nutritional Risk Screening 2002; OR, odds ratio; PDFF, proton density fat fraction; rRNA, ribosomal ribonucleic acid; SANRA, Scale for the Assessment of Narrative Review Articles; SARC-F, Strength, Assistance in walking, Rise from a chair, Climb stairs, and Falls questionnaire; UK, United Kingdom.
